# Identification of genetic factors governing drought tolerance in wild and domesticated tetraploid wheat

**DOI:** 10.1186/s12864-026-13221-9

**Published:** 2026-08-01

**Authors:** Santosh Gudi, Jatinder Singh, Justin Faris, Upinder Gill, Rajeev Gupta

**Affiliations:** 1 Department of Plant Pathology, Microbiology and Biotechnology, North Dakota State University, Fargo, ND USA; 2 Cereal Crops Improvement Research Unit, Edward T. Schafer Agricultural Research Center, USDA-ARS, Fargo, ND USA

**Keywords:** Chromosome substitution lines, Drought tolerance, Durum wheat, QTL mapping, Recombinant inbred lines, Wild emmer wheat

## Abstract

Germplasm characterization and gene identification are essential first steps in developing drought-resilient cultivars. Evaluation of diverse tetraploid wheat accessions representing cultivated and wild species under severe drought stress using an *in-house* protocol identified three extremely tolerant genotypes including the CIMMYT-bred durum (*Triticum turgidum* ssp. *durum*) variety Altar 84 and the two wild emmer (*T. turgidum* ssp. *dicoccoides*) accessions PI 478742 and PI 481521. Chromosome substitution lines involving individual pairs of chromosomes from PI 478742 and PI 481521 substituted for homologous pairs of chromosomes in drought sensitive durum variety Langdon 16 were evaluated to identify chromosome(s) carrying loci controlling drought tolerance. The 11 chromosome substitution lines available for PI 478742 (2A, 3A, and 3B substitution lines were not available) were all sensitive to drought suggesting tolerance may be conferred by a locus on one of the chromosomes not evaluated. All 14 possible chromosome substitution lines were available for PI 481521, and among these, 13 were drought sensitive. The substitution line involving chromosome 3A (LDN-DIC 3A(521)) was tolerant to drought. QTL mapping in a durum inter-varietal population of 138 recombinant inbred lines (RILs) derived by crossing Altar 84 and Langdon 16 identified a major QTL on the short arm of chromosome 4B explaining 26% of phenotypic variation (PVE). This QTL spans large physical region of approximately 265.52 Mb in the Svevo RefSeq v1.0 genome. Sequence analysis of *TRITD4Bv1G024340*, an orthologue of drought tolerance gene (*TaWD40-4B.1*) cloned and previously reported from same genomic region in hexaploid wheat, suggested that QTL identified in this study in Altar 84 is governed by a different genomic region. Additionally, two drought tolerance regions identified (on chromosome 3A and 4BS) in this study are two independent QTLs originated from different sources, and hence they might have different genetic tolerance mechanism. Drought tolerant lines and the genomic regions identified in this study serve as valuable resources for developing drought-resilient durum wheat varieties.

**Supplementary Information:**

The online version contains supplementary material available at https://doi.org/10.1186/s12864-026-13221-9.

## Introduction

Durum wheat (*T. turgidum* L. ssp. *durum* (Desf.) van Slageren, 2*n* = 4*x* = 28, AABB genomes) is an allotetraploid, that accounts for 5–8% of wheat production worldwide [1]. It is an economically important crop because of its unique grain quality traits that make it suitable for making pasta and other semolina-based products. Durum wheat is primarily produced in Canada and the USA with global annual production of ~ 1.3 billion bushels ( wmr-world-durum-2024 ). In the USA, North Dakota and Montana are the leading producers, accounting for more than 80% of the country’s durum wheat production (https://www.ndwheat.com/durum-wheat). Durum wheat production is severely affected by various biotic and abiotic stresses, including drought, heat, salinity, lodging, etc [2–8]. Among these, drought is one of the top three environmental stresses, along with cyclones and severe storms, causing an estimated annual loss of ~$6–9 billion in the USA. For instance, the severe drought stress of 2012 in the USA affected ~ 80% of agricultural land, significantly reducing the production of livestock and major field crops including wheat, corn and soybean, resulting in an estimated economic loss of ~$14.5 billion (https://www.drought.gov/sectors/agriculture).

Drought stress affects agronomic, morpho-physiological, molecular, and biochemical traits at various developmental stages of wheat. It significantly decreases leaf relative water content (RWC) and CO_2_ assimilation rate, thereby reducing photosynthetic efficiency and biomass accumulation [9, 10]. Drought stress results in excessive accumulation of reactive oxygen species (ROS), which disrupts critical biochemical and metabolic processes of cells [11]. The impact and severity of drought stress depend on its intensity (continuous drought, intermittent drought, or once per season), duration (short or long), and the developmental stage (vegetative or reproductive) of the crop [12]. During the vegetative stage, drought stress inhibits root length, root hairs, leaf area, plant height, and tiller number, which ultimately reduces grain yield per unit area [13–16]. However, drought stress during reproductive and grain filling stages cause yield losses up to 50–60% by reducing harvest index, spike length, spikelet number per spike, grains number per spike, grain length, grain width, 1000-grain weight, and grain filling period [17–21]. The impacts of drought stress are expected to escalate in the future due to global warming coupled with unpredictable and erratic rainfall patterns. Therefore, it is necessary to develop drought resilient durum wheat cultivars to overcome the impacts of drought stress as well as to ensure global food security. First step to achieve this is characterization of diverse germplasm lines for drought tolerance. Multiple studies used adult plant screening (i.e., at anthesis and grain filling stages) under field conditions for drought tolerance in wheat [15, 22–24]. Though, evaluation at later developmental stages is the right approach, it is constrained by: (i) lack of control on environmental variabilities; (ii) characterization of limited number of germplasm lines; and (iii) required large scale field studies. Considering limited genetic diversity for drought tolerance and requiring evaluation of large number of germplasm collections, we need to develop a quick and reliable screening methods which can facilitate evaluation of thousands of germplasm lines in short time. Seedling stage evaluation is the favorite for initial germplasm screening, and several studies used it to identify source of drought tolerant genotypes in wheat [20, 25, 26]. These studies reported high correlation between seedling stage drought tolerance and grain yield and showed that seedlings subjected to water deficit could be used as an indicator of drought tolerance in adult plants under field conditions in wheat [27, 28], triticale [29], and maize [29, 30].

Historically, wheat breeding programs focused primarily on developing cultivars with improved grain yield. While doing this, much allelic variation for disease resistance and stress resiliency was removed unintentionally from the crop gene pools [1, 31]. Additionally, two successive domestication events reduced the genetic diversity of cultivated durum wheat by ~ 85% compared to their wild relatives [1]. The first event occurred around ~ 12,000 years ago in the Fertile Crescent, which resulted in the replacement of brittle type of wild emmer wheat to a non-brittle type of cultivated emmer wheat. A second event occurred ~ 10,000 years ago, where further domestication of emmer forms into the modern free-threshing durum wheat [32–34]. The narrow genetic base in gene pool makes cultivated wheat varieties extremely vulnerable to diseases, pests, and environmental stresses [35, 36]. One effective way to restore this lost genetic variation is the introduction of unexplored landraces, wheat progenitors, and the wild wheat relatives (WWRs) into breeding programs, as they represent a valuable resource for genetic diversity and beneficial alleles [37, 38]. Over the last few decades, various wild and cultivated tetraploid wheat accessions have served as sources of stress resiliency in durum wheat. Some of them include wild emmer wheat (*T. turgidum* ssp. *dicoccoides* (Körn. ex Asch. & Graebn.) Thell.) [39, 40], cultivated emmer wheat (*T. turgidum* ssp. *dicoccum* (Schrank) Thell.) [41], and Persian wheat (*T. turgidum* ssp. *carthlicum* (Nevski) Á. Löve & D. Löve) [42]. Wild emmer wheat, a tetraploid progenitor of domesticated wheat, originated from the eastern Mediterranean region [43, 44], and the majority of wild emmer wheat germplasm has been collected from Israel, Syria, Iran, Iraq, and Turkey, which are characterized by a hotter and drier climate with fluctuating amounts and distribution of rainfall [45]. As a result, wild emmer wheat has developed high levels of drought tolerance and likely harbors a rich allelic repertoire for drought stress tolerance [46, 47]. Previous studies characterized several cultivated and wild tetraploid wheat accessions for drought stress tolerance [23, 41, 42, 48]. For instance, Peleg et al. [47] evaluated 110 wild emmer wheat accessions under normal and drought stress and identified promising lines with higher productivity under drought. Similarly, Bacher et al. [40] assessed wild emmer wheat introgression lines under drought stress and identified an introgression line with significant shift in root-to-shoot ratio. These studies highlighted the genetic potential of wild emmer wheat for improving drought stress tolerance.

Evaluating genetic stocks and mapping populations can facilitate locating genes responsible for targeted traits. Genetic stocks widely used in durum wheat are chromosome substitution lines, which have a specific chromosome from a donor parent in the background of recipient parent. During the 1980s, Dr. Leonard R. Joppa (USDA-ARS, Fargo, ND, retired) developed three sets of Langdon 16 durum-*T. turgidum* ssp. *dicoccoides* (LDN-DIC) substitution lines using wild emmer wheat accessions Israel A, PI 481521 (LDN-DIC (521)) and PI 478742 (LDN-DIC (742)) as donors [49, 50]. These substitution lines are isogenic to Langdon 16 except for the substituted chromosome. Therefore, any phenotypic differences observed between Langdon 16 and a substitution line can be attributed to genes present on the substituted wild emmer chromosome. Multiple studies used the substitution lines to locate genes associated with various traits including grain protein content (GPC) [51, 52], glutenin subunits and gliadins [53, 54], kernel characteristics and protein molecular weight distribution [55], disease resistance [56, 57], threshability [58], spike morphology [59], glaucousness/waxiness [60], and grain yield related traits [51]. However, these genetic stocks have not yet been systematically evaluated for drought tolerance, giving an opportunity to assess their performance under drought stress conditions.

The genetics of drought stress tolerance is complex and quantitative in nature, typically controlled by multiple genes with minor effects. Additionally, drought stress is strongly influenced by various environmental factors, making genetic studies complicated. The advent of molecular markers facilitated precise mapping of complex traits through quantitative trait loci (QTL) and genome-wide association studies (GWAS) [61]. Several studies utilized these techniques to identify and characterize key genes associated with drought stress tolerance in hexaploid wheat. Some of them include *TaNAC071-A* [62], *TaPYL1-1B* [63], *DIW1/TaPP2C158* [64], *TaWD40-4B.1* [65], *TabHLH27* [66], *TaABF2* [67], *TaPYL9* [68], *TaCLE24b* [69], *TaDT1-A* [70], and *TaLBD1* and *TaGLYI7* [71]. In contrast, studies focused on the identification of drought tolerance QTLs in tetraploid wheat are limited, with only a few notable examples [39, 72, 73]. Furthermore, studies on the functional validation of drought tolerance genes in tetraploid wheat are scarce with only a few genes, *TdicTMPIT1* [74], *TdicDRF1* [75], and *TdATG8* [76], having been examined using qRT-PCR. Despite these efforts, most of the identified QTLs/genes has not been successfully integrated into breeding programs, as they tend to be unstable across environments and exhibit minor phenotypic effects.

In this study, we screened diverse accessions of cultivated and wild tetraploid wheat under drought stress to identify drought-tolerant genotypes. Chromosome substitution lines were evaluated to identify a chromosome from wild emmer harboring factors conferring drought tolerance, and a recombinant inbred line (RIL) was used to map genomic regions associated with drought stress tolerance in durum wheat. The drought tolerant genotypes and the genomic regions identified in this study may serve as valuable resources for developing drought resilient durum wheat cultivars.

## Materials and methods

### Plant materials

In this study, we screened 19 diverse tetraploid wheat accessions belonging to *T. turgidum* ssp. *durum* (12 accessions), *T. turgidum* ssp. *dicoccum* (2 accessions), *T. turgidum* ssp. *dicoccoides* (3 accessions), and *T. turgidum* ssp. *carthlicum* (2 accessions) (Supplementary Table 1) for response to drought. Two sets of LDN-DIC chromosome substitution lines developed by substituting the chromosomes of *T. turgidum* ssp. *dicoccoides* accessions PI 478742 and PI 481521 for the homologous pair of chromosomes in the durum variety Langdon 16 (LDN) were also evaluated. These chromosome substitution lines were developed by the late Dr. Leonard R. Joppa at the USDA-ARS laboratory in Fargo, ND, and have been described in Xu et al. (2004). The set with donor chromosomes derived from PI 481521 (LDN-DIC (521)) consists of all 14 possible chromosomal substitutions. In contrast, the PI 478742 derived set (LDN-DIC (742)) consists of only 11 of the possible 14 substitution lines because Dr. Joppa was unable to obtain substitutions involving chromosomes 2 A (LDN-DIC 2 A(742)), 3A (LDN-DIC 3A(742)), and 3B (LDN-DIC 3B(742)). We also evaluated a population of 138 recombinant inbred lines (RILs) derived by crossing Langdon 16 and Altar 84 (hereafter referred to as AL-RIL population) (Supplementary Table 1). This population was developed by advancing F_2_ plants to the F_7_ generation using the single seed descent (SSD) method [77].

### Experimental design and phenotypic evaluation

**Fig. 1:**
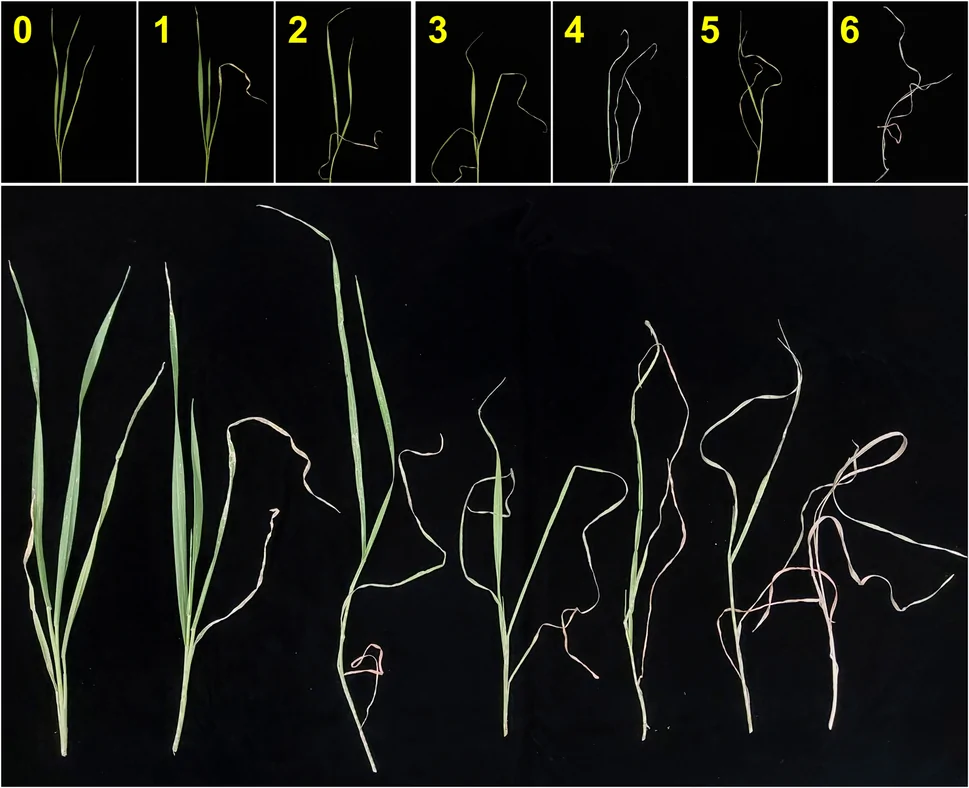
Wilting score (WS) scale used for phenotypic data collection under drought stress. “WS 0” means extremely drought tolerance and “WS 6” means extremely drought sensitive

All experiments were conducted at the USDA-ARS Northern Crop Science Laboratory greenhouse facility at Fargo, North Dakota, USA (46.9002° N latitude, 96.8032° W longitude). Greenhouse conditions were set to 16-hour photoperiod (lux) with 25 °C day temperature and 22 °C night temperature. Relative humidity was maintained at 65%. A description of the protocol for drought stress is provided in Supplementary Fig. 1. In brief, a total of 42 uniform and bold seeds from each genotype were disinfected with 10% Clorox for 20 min, followed by rinsing three times with deionized water (diH_2_O) for 5 min each. Disinfected seeds were kept at 4 °C for 48 h to overcome dormancy or to induce uniform seed germination. Before sowing, pots (with dimension of 10.5 cm L × 10 cm W × 12 cm H) were filled with 200 g of soil (PRO-Mix BRK20 Biofungicide + Mycorrhizae peat/bark mix), and 300 ml of water was added to each pot to maintain uniform moisture conditions across the experiment. Seven seeds from each genotype were sown in six pots (i.e., 7 × 6 = 42 seeds). The experiment followed a randomized complete block design (RCBD), with two pots per genotype constituting one replication, and hence six pots assigned to three replications (six pots per genotype). To minimize environmental variations caused by greenhouse border effects such as variation in air movement and temperature, border rows were maintained by planting a wheat genotype (Langdon 16) in pots of the same size as those used in the main experiment (Supplementary Fig. 2). Upon germination (usually 3–4 days after sowing), seedlings were thinned by maintaining only five seedlings in each pot leaving a total of 10 seedlings per genotype per replication. An equal amount of fertilizer (5 gm) (Osmocote Plus 15-9-12 patterned release fertilizer), and 100 ml water was added to each pot after thinning. Subsequently, 100 ml water was added to each pot every alternative day until two-leaf stage (up to approximately 10–12 days after sowing). Drought stress was initiated at the two-leaf stage (after adding 100 ml water) by withholding water, and the gravimetric soil moisture content was monitored using soil moisture meters (Zynect Soilmote Sensors). Drought stress was applied until the gravimetric soil moisture content fell below 5% and some genotypes began to show wilting symptoms (i.e., usually 20–22 days). Once the drought stress period was complete, pots were rewatered to full saturation and allowed to recover for two days. Data on wilting score (WS) was recorded based on the top three leaves using a 0–6 scale, which included minor modifications of the scale suggested by Tian et al. [65] (Fig. 1). “WS 0” means no wilting symptoms with all three of the top leaves being healthy; “WS 1” means the top two leaves were healthy with partial wilting of the third leaf; “WS 2” means the top two leaves were healthy with complete wilting of the third leaf; “WS 3” means the top leaf was healthy with partial wilting of the second leaf and complete wilting of the third leaf; “WS 4” means the top leaf was healthy with complete wilting of the second and third leaves; “WS 5” means partial wilting of the top leaf and complete wilting of the second and third leaves; and “WS 6” represented a completely wilted or dead plant. Genotypes with less than five seedlings per pot or those having one or more weak seedlings in a pot were excluded from the study to avoid potential bias in the results.

### Statistical analysis

Raw phenotypic data on seedling wilting score were subjected to mixed linear analysis to derive best linear unbiased prediction (BLUP) with LME4 package using the following model:

$$\mathrm{Y}_{\mathrm{ab}}=\upmu+\mathrm{G}_{\mathrm{a}}+\mathrm{R}_{\mathrm{b}}+\mathrm{e}_{\mathrm{ab}}$$

Where, Y_ab_ is the seedling wilting score, µ is the overall mean, G_a_ is the effect of a^th^ genotype, R_b_ is the effect of b^th^ replication, e_ab_ is the error associated with a^th^ genotype and b^th^ replication, which is assumed to be normally and independently distributed, with mean zero and homogeneity of variances as V.

Broad-sense heritability (h^2^_bs_) for was calculated by using the following formula:

$$\:{{\mathrm{h}}_{\mathrm{bs}}}^{2}=\frac{Vg}{\mathrm{V}\mathrm{g}+\mathrm{V}\mathrm{e}/R}$$

Where, h _bs_^2^ is the broad-sense heritability, Vg is the genetic variance, Ve error variance, R is the number of replications.

### Marker genotyping, linkage map construction and QTL mapping

Genotyping and linkage analysis of the AL-RIL population was conducted by Virdi et al. [77]. In brief, the AL-RIL population was genotyped using the iSelect 9 K single nucleotide polymorphism (SNP) array [78] and simple sequence repeat (SSR) markers selected from WMS (gwm) [79], WMC [80], HBG [81], CFD [82], BARC [83], and MAG [84] libraries. A total of 843 markers (including SNP and SSR) were used for linkage map construction.

Composite interval mapping (CIM) was employed for QTL detection using the software program QGENE 4.3.10v [85]. The WS data from individual replications, mean values, and BLUP values were used as phenotypic data. The LOD threshold for QTL significance was determined by conducting a permutation test with 1000 iterations at the 0.05 level of significance. The additive effects of QTLs and the coefficient of determination (*R*^2^) were calculated to determine the effect and the amount of phenotypic variation explained by each QTL, respectively. Nucleotide sequences of the markers identified in QTL region were retrieved from GrainGenes database (https://wheat.pw.usda.gov/) and were subjected to BLASTn searches against Svevo RefSeq v1.0 [1] and Chinese Spring RefSeq v2.1 [86] to delineate the physical positions of markers.

### DNA extraction and gene sequencing

DNA from young and healthy leaves of Altar 84 and Langdon 16 were extracted using QIAGEN’s DNeasy Plant Pro Kit following the manufacturer’s instructions (https://www.qiagen.com). RNA contamination was removed by treating DNA with Thermo Scientific RNase A at 37 °C for 20 min. Quantity and quality of DNA was assessed using NanoDrop 8000 spectrophotometer.

The complete gene sequence of *TRITD4Bv1G024340*, a homologue of the hexaploid wheat gene *TaWD40.4B.1*, including 200 bp upstream and downstream regions (~ 2300 bp), was amplified from Altar 84 and Langdon 16 using primer pairs P1_F (forward primer) and RP6 (reverse primer) in three replications (Supplementary Table 2). PCR was carried out in 50 µl reaction involving 20 ng/µl of template DNA, 5 µl of 10x LA PCR buffer II, 5 µl of 25 mM MgCl_2_, 8 µl of dNTP mixture, 1 µl of each primer, and 0.5 µl of TaKaRa LA Taq DNA polymerase (Takara Bio Inc., Kusatsu, Shiga, Japan). PCR was carried out using an Applied Biosystems MiniAmp Thermal Cycler (Thermo Fisher Scientific, Waltham, Massachusetts, USA) with initial denaturation at 94 °C for 5 min followed by 35 cycles of denaturation (i.e., 98 °C for 20 s), annealing (i.e., 58.4 °C for 45 s), extension (i.e., 68 °C for 2 min), followed by final extension at 72 °C for 10 min. An annealing temperature of 58.4 °C was chosen based on a gradient PCR test with temperatures ranging from 55 °C to 64 °C (Supplementary Fig. 3a). PCR amplification was confirmed from 10 µ product from each sample in 1.5% agarose gel electrophoresis (Supplementary Fig. 3b). The remaining 40 µl of PCR product was purified using ExoSAP IT (Thermo Fisher Scientific, Waltham, Massachusetts, USA), and samples were sent for Sanger sequencing (Eurofins Genomics, Louisville, KY, USA). Sanger sequencing was done using P1_F and RP6 primers along with three internal primers (P1_R, FP625bp, and FP1281bp). All primer sequences were designed de-novo from the durum wheat reference sequence (Svevo RefSeq v1.0) using the NCBI primer-design tool (https://www.ncbi.nlm.nih.gov/tools/primer-blast/) (Supplementary Table 2). Sequencing reads were assembled into consensus gene sequences for both lines using Geneious software (https://www.geneious.com/). Consensus gene sequences were subjected to BLAST searches against the *TaWD40.4B.1* gene to identify point mutations in Altar 84 and Langdon 16. Furthermore, sequences from drought tolerant (Altar 84) and drought sensitive (Langdon 16) genotypes were compared to identify polymorphisms in the *TaWD40.4B.1* gene sequence.

## Results

### Response of wheat lines to drought stress

**Fig. 2:**
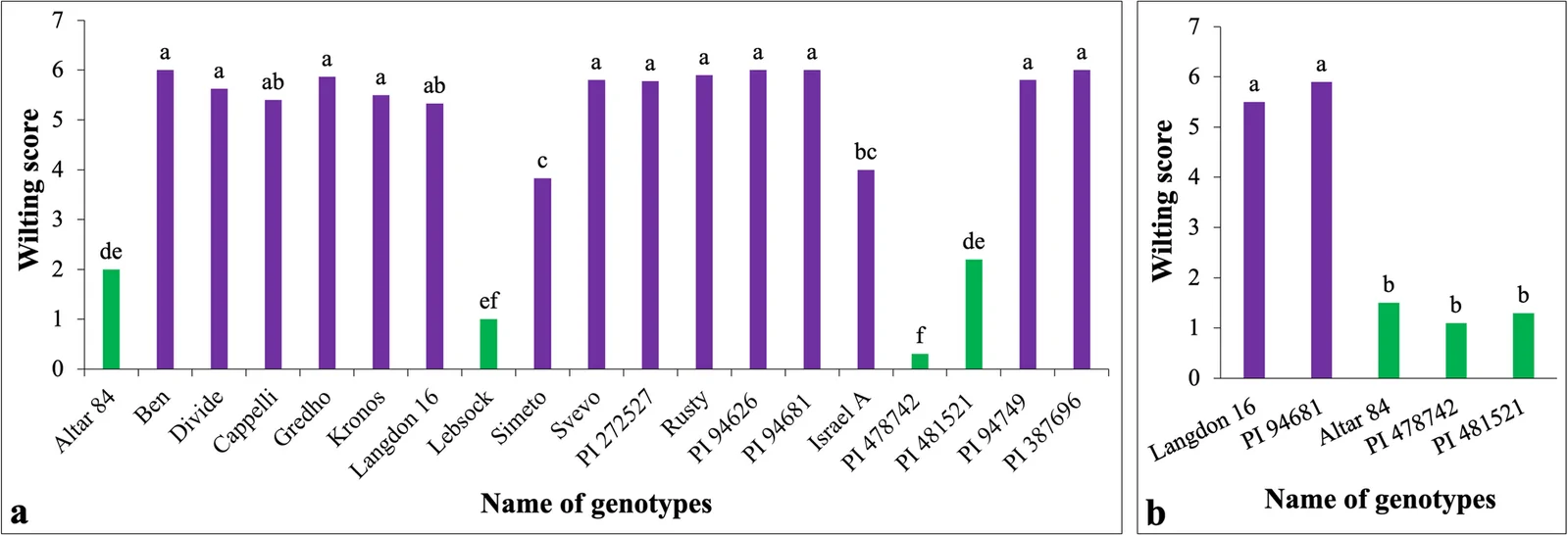
Bar graph showing the phenotypic evaluation of: (**a**) diverse tetraploid wheat accessions and (**b**) contrasting genotypes (Langdon 16, PI 94681, Altar 84, PI 478742, and PI 481521) under extreme drought stress. Green bars indicate WS < 3 and were considered drought-tolerant lines. Purple bars indicate WS > 3 and were considered drought-sensitive lines. Different letters above the bars indicate statistically significant differences among the lines based on least significant difference (LSD) (*P* < 0.05)

The 19 tetraploid wheat lines evaluated showed significant (*P* < 0.001) variation for wilting score (WS) with 18.65% coefficient of variance (CV) and an LSD of 1.42 (Table 1). Thirteen lines were highly sensitive to drought stress with WS > 5 and completely dead after 20 days of drought stress. Two lines were moderately tolerant (WS = 3–5), and the remaining four genotypes were tolerant with WS < 3 (Fig. 2a). Among the tolerant genotypes, the *T. turgidum* ssp. *dicoccoides* accession PI 478742 was extremely tolerant to drought stress with a WS of 0.3. The remaining three drought tolerant lines included the durum varieties Lebsock (WS = 1) and Altar 84 (WS = 2), and the wild emmer accession PI 481521 (WS = 2.2) (Supplementary Table 3).

**Table 1:** Descriptive statistical analysis for wilting score (WS) in various populations evaluated in this study

Genotypes/population	Number of genotypes	F-value	Pr(> F)	CV (%)	H^2^_bs_ (in %)
Parental lines	20	7.457	1.30E-06***	20.59	89.7
Contrasting lines	6	36.11	2.35e-05***	18.43	97.6
LDN-DIC (742)	13	9.89	1.86e-06***	11.2	92.3
LDN-DIC (521)	16	9.54	1.18e-07***	14.73	89.5
RILs	140	3.65	2.2e-16 ***	22.3	72.6

*RILs* recombinant inbred lines, *CV* coefficient of variance, *H*^*2*^_*bs*_ broad-sense heritability

Level of significance: *p* < 0.05 (*); *p* < 0.01 (**); *p* < 0.001 (***)

To confirm the drought responses of the four tolerant lines, we evaluated them in a second experiment along with two sensitive genotypes (Langdon 16 and PI 94681) (Supplementary Table 4). PI 478742, PI 481521, and Altar 84 showed a consistent drought tolerance response (Fig. 2b), whereas Lebsock was sensitive in the second experiment.

### Evaluation of LDN-DIC (742) chromosome substitution lines under drought stress

**Fig. 3:**
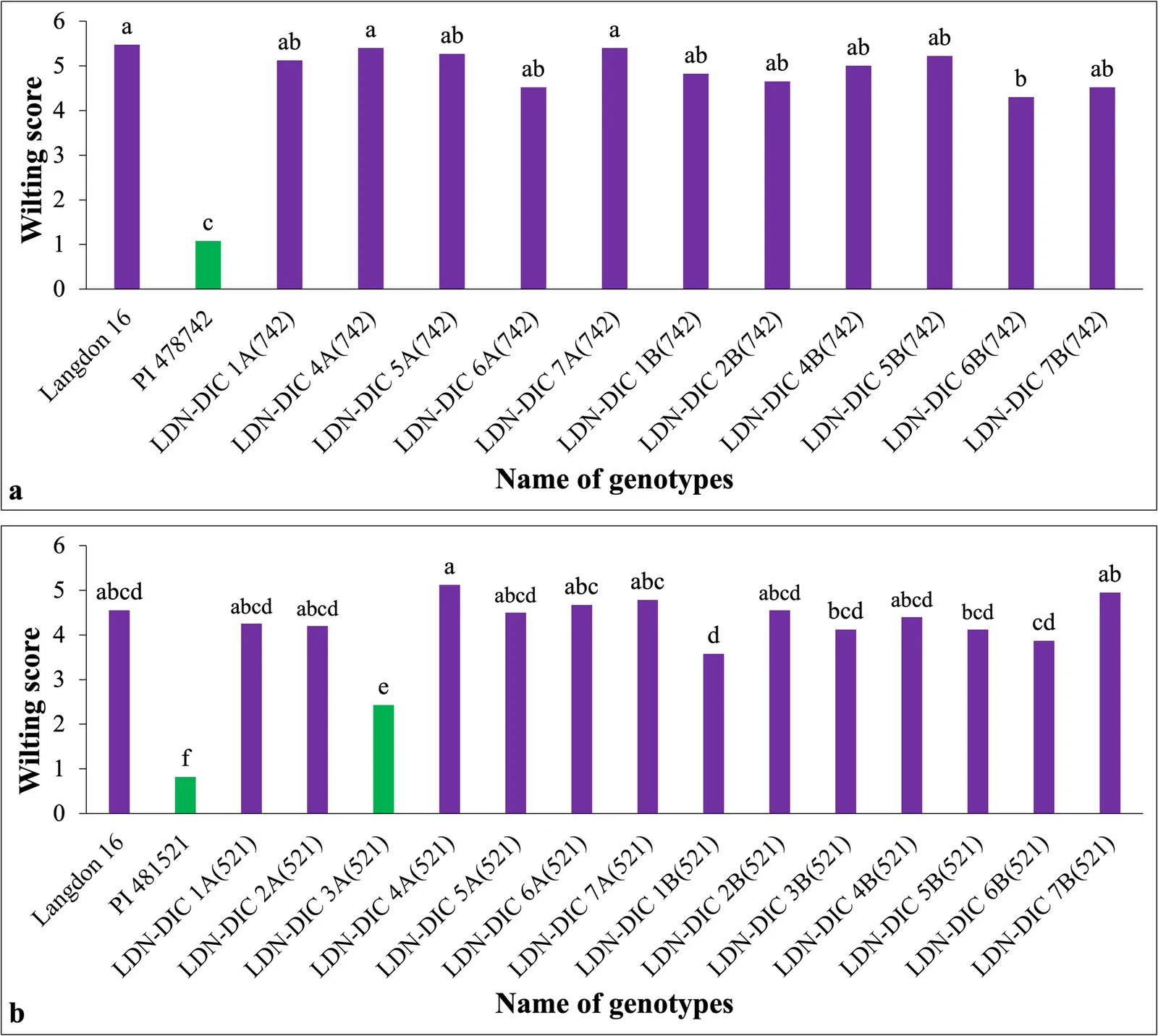
Phenotypic evaluation of: (**a**) Langdon 16, the *Triticum turgidum* ssp. *dicoccoides* accession PI 478742, and the chromosome substitution lines (LDN-DIC (742)) with PI 478742 chromosomes substituted for homologous Langdon 16 chromosomes; and (**b**) Langdon 16, the *Triticum turgidum* ssp. *dicoccoides* accession PI 481521, and the chromosome substitution lines (LDN-DIC (521)) with PI 481521 chromosomes substituted for homologous Langdon 16 chromosomes. Green bars indicate WS < 3 and were considered drought-tolerant lines. Purple bars indicate WS > 3 and were considered drought-sensitive lines. Different letters above the bars indicate statistically significant differences among the lines based on least significant difference (LSD) (*P* < 0.05)

Evaluation of the LDN-DIC (742) chromosome substitution lines resulted in an average WS for all 11 LDN-742 substitution lines not significantly different from Langdon 16 (i.e., 5.47) (Fig. 3a; Supplementary Table 5). However, substitution lines for chromosomes 2A (LDN-DIC 2 A(742)), 3A (LDN-DIC 3A(742)), and 3B (LDN-DIC 3B(742)) were not available. Therefore, we speculate that the drought tolerance in PI 478742 may be conferred by factors on one or more of these three chromosomes 2A, 3A, and 3B.

### Evaluation of LDN-DIC (521) chromosome substitution lines located drought tolerance gene(s) on chromosome 3A

**Fig. 4:**
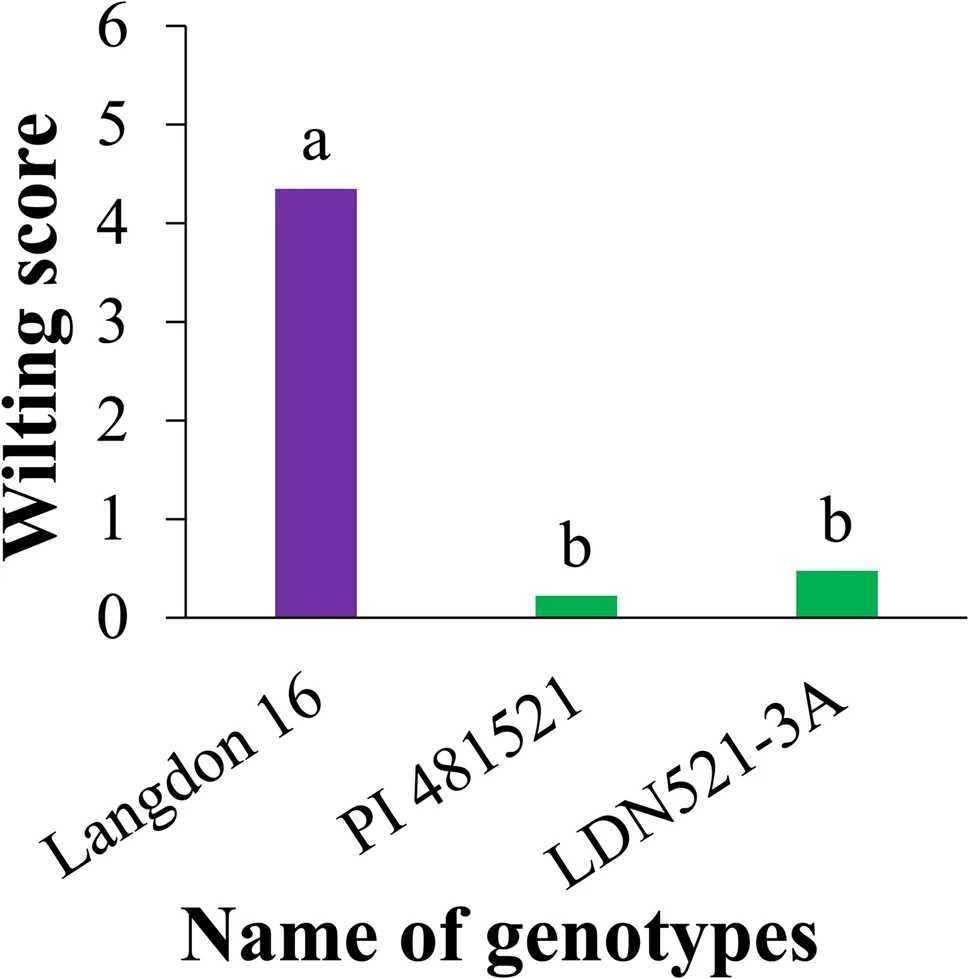
Phenotypic evaluation of Langdon 16 (drought sensitive parent), PI 481521 (drought tolerant parent) and LDN-DIC 3A(521) (drought tolerant substitution line) under drought stress in a second experiment. Different letters above the bars represent significant differences based on least significant difference (LSD) (*P* < 0.05)

Evaluation of the LDN-DIC (521) substitution lines indicated that 13 were drought-sensitive. However, substitution line LDN-DIC 3A(521) was tolerant to drought with a WS of 2.43 (Fig. 3b; Supplementary Table 6). To further confirm the drought tolerance of LDN-DIC 3A(521), we evaluated LDN-DIC 3A(521), Langdon 16, and PI 481521 in six additional replications. These results confirmed that LDN-DIC 3A(521) had a high level of drought tolerance with a WS = 0.5, which is comparable to its donor parent, PI 481521, which had a WS of 0.2 (Fig. 4; Supplementary Table 7). From these results we confirmed that the drought tolerance gene(s) in PI 481521 are located on chromosome 3A.

### Evaluation of the AL-RIL population identified a drought tolerance QTL on the short arm of chromosome 4B

**Fig. 5:**
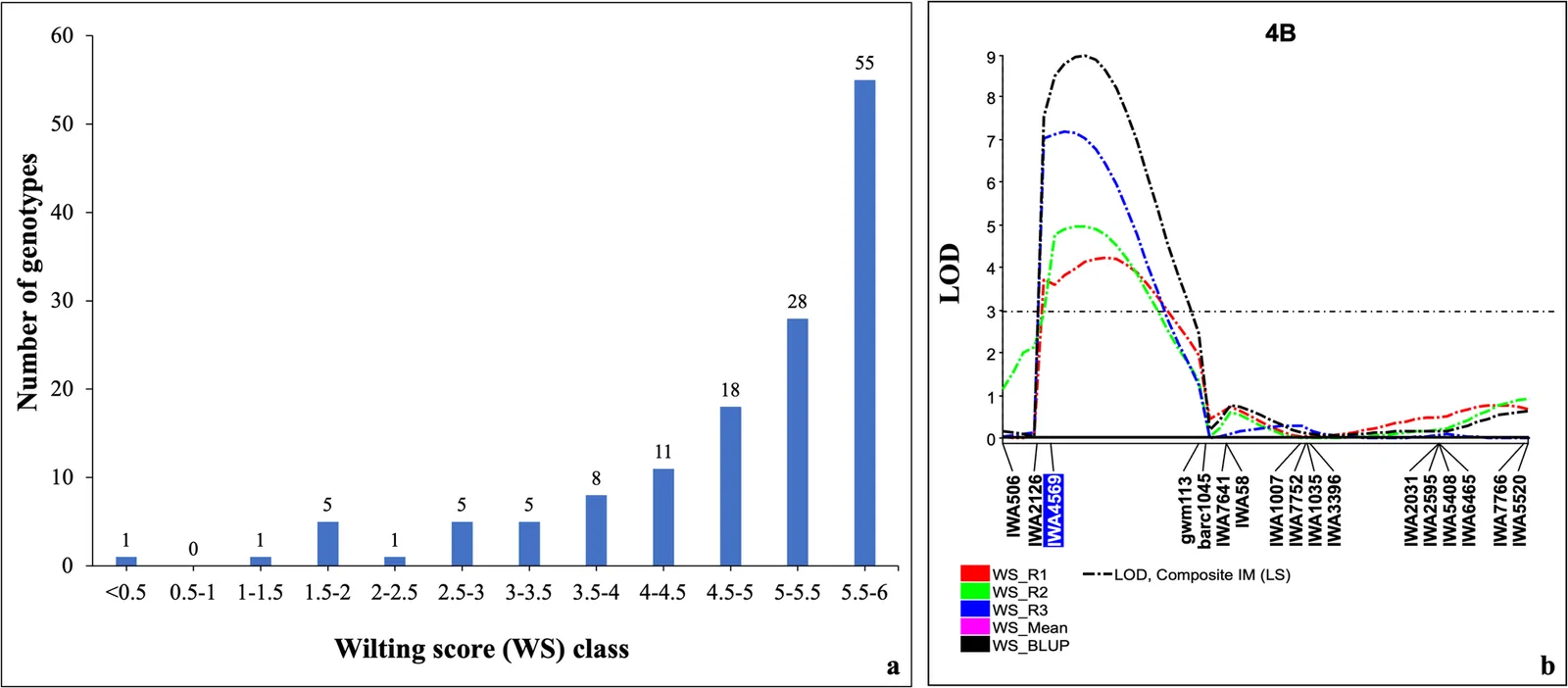
**a** Phenotypic evaluation and (**b**) quantitative trait loci (QTL) mapping for drought stress tolerance in the recombinant inbred line (RIL) population derived from Altar 84 (drought tolerant parent) and Langdon 16 (drought sensitive parent). A dashed line indicates a LOD cutoff of 3.0. Dotted lines along the graphs represent the results for replication 1 (WS_R1; red), replication 2 (WS_R2; green), replication 3 (WS_R3; blue), mean WS data (WS_mean; pink), BLUP values (WS_BLUP; black). A marker highlighted in blue color represents forward cofactor selection in composite interval mapping (CIM) method. WS: wilting score; BLUP: best linear unbiased prediction

To identify the genetic factors responsible for drought tolerance in Altar 84, we used AL-RIL population developed by crossing Langdon 16 and Altar 84. Most RILs in the AL population were as sensitive to drought as Langdon 16. However, 18 RILs were tolerant to drought stress (Fig. 5a; Supplementary Table 8). Analysis revealed a single QTL (designated as *QDt.fcu-4B*) significantly associated with drought tolerance (i.e., WS) (Fig. 5b; Supplementary Fig. 4). *QDt.fcu-4B* was located on short arm of chromosome 4B between markers *IWA506* and *barc1045*, which were separated by ~ 40.27 cM. Based on mean WS, *QDt.fcu-4B* had a LOD score of 9 and explained 26% phenotypic variation (PVE). The allele contributing to drought tolerance at this locus was derived from Altar 84. Consequently, individuals carrying the drought tolerant allele exhibit lower WS compared to those lacking this allele. Additionally, *QDt.fcu-4B* independent of that identified on chromosome 3A, and it may have different drought tolerance mechanism.

To delineate the physical position of *QDt.fcu-4B*, we retrieved the nucleotide sequences of markers flanking the QTL region. The marker *IWA506* was located at the position 9,123,607-9,123,727 bp in Svevo RefSeq v1.0 and at 11,540,771 − 11,540,891 bp in Chinese Spring RefSeq v2.1. In contrast, the physical position of *barc1045* was identified only in Svevo RefSeq v1.0 at the position 274,641,313 − 274,641,333 bp. These results indicate that *QDt.fcu-4B* occupies a large genomic region of approximately 265.52 Mb. The large QTL interval identified in this study is likely a consequence of low marker density used for genetic mapping, which reduced the QTL resolution. Consequently, this QTL must be regarded as large effect but low-resolution locus suitable for preliminary genetic characterization.

### *QDt.fcu-4B* is a unique QTL

Several QTLs and GWAS studies have reported the presence of drought tolerance QTLs on short arm of chromosome 4B, making it a genomic hotspot for drought stress tolerance in wheat. Moreover, Tian et al. [65] cloned a drought tolerance gene, *TaWD40-4B.1* (QTL region spanning 66.8–68.4 Mb as per Chinese Spring RefSeq v1.0.), from this region in hexaploid wheat (*T. aestivum*). The drought tolerance QTL, *QDt.fcu-4B*, identified in our study colocalized with this region, raising the possibility that the drought tolerance locus in Altar 84 might be governed by *TaWD40-4B.1*. To test this hypothesis, we retrieved the nucleotide sequence of *TaWD40-4B.1* (*TraesCS4B03G0161800*; 70,456,229 − 70,458,157 bp from Chinese Spring RefSeq v2.1 or *TraesCS4B02G072200*; 67,352,463 − 67,354,391 bp Chinese Spring RefSeq v1.0) and conducted BLAST alignments against Svevo RefSeq v1.0. This resulted in the identification of ~ 2 Kb orthologous gene, *TRITD4Bv1G024340* (65,955,068 − 65,956,996 bp), with 100% sequence identity and full coverage.

Comparison of consensus gene sequences of Altar 84 and Langdon 16 with the functional allele of *TaWD40-4B.1* identified four SNPs at 631 bp (A to G), 1165 bp (T to C), 1440 bp (G to A), and 1623 bp (A to G) in both lines (Supplementary Fig. 5a, b). The non-sense mutation (SNP1274) at 1440 bp (G to A) identified by Tian et al. [65] was present in both lines, indicating that both lines carry the drought sensitive allele of the *TaWD40-4B.1* gene. The two synonymous SNPs (SNP465 and SNP999) located at 731 bp (G to A) and 1164 bp (T to C), respectively, identified by Tian et al. [65] were missing in these lines. However, two SNPs at 631 bp and 1165 bp were not present in hexaploid wheat and hence they were unique to tetraploid wheat. Moreover, gene sequences of Altar 84 and Langdon 16 were 100 percent identical (Supplementary Fig. 5c).

## Discussion

Changing weather patterns coupled with unpredictable and erratic rainfall are inducing severe drought stress and are primary concerns for sustainable production in major durum wheat growing countries. To overcome the negative impacts of drought stress, it is crucial to identify and introgress alleles that confer drought tolerance. However, the genetic diversity for drought tolerance has been eroded in the cultivated durum wheat gene pool due to crop domestication and stringent artificial selection for grain yield related traits [1]. One option for the reintroduction of genetic diversity is to explore the natural diversity present among the wheat progenitors. To achieve this, we evaluated tetraploid wheat accessions belonging to *T. turgidum* ssp. *durum*, *T. turgidum* ssp. *dicoccum*, *T. turgidum* ssp. *dicoccoides*, and *T. turgidum* ssp. *carthlicum* under severe drought stress. This study resulted in the identification of extremely drought tolerant CIMMYT-bred durum variety Altar 84 and two wild emmer (*T. turgidum* ssp. *dicoccoides*) accessions PI 478742 and PI 481521. Wild emmer wheat is a valuable source of drought tolerance genes. Previous studies identified several drought tolerant accessions of wild emmer wheat and reported key genes, such as *TdicTMPIT1* [74], *TdicDRF1* [75], and *TdATG8* [76], responsible for improving drought tolerance in wild emmer wheat.

### Chromosome substitution lines mapped drought tolerance to chromosome 3A from wild emmer accession PI 481521

Chromosome substitution lines are the genetic stocks that help in identifying the chromosomes carrying gene of interest. Evaluation of LDN-DIC (742) set under drought stress revealed that drought tolerance in PI 478742 is present on either of the three chromosomes 2A, 3A, or 3B. To further investigate this, we are currently developing a RIL population by crossing Langdon 16 and PI 478742 to map the gene responsible for drought stress tolerance in PI 478742. Similarly, evaluation of the LDN-DIC (521) set revealed that only one of the 14 substitution lines, LDN-DIC 3A(521), displayed high drought tolerance. These results suggested that drought tolerance gene in PI 481521 is located on the chromosome 3A. Because we identified drought tolerance derived from chromosome 3A of PI 481521, it is possible that drought tolerance in PI 478742 is also controlled by genes located on 3A. These results are consistent with previous studies reporting the stable and pleiotropic QTL on chromosome 3A responsible for improving photosynthetic and water use efficiency traits under drought stress [87, 88]. More interestingly, analysis of chromosome substitution lines of Cheyenne and Hope in the background of Chinese Spring identified chromosome 3A substituted lines with improved ABA responsiveness and stomatal regulation during leaf dehydration [89]. Similarly, evaluation of two sets of reciprocal chromosome substitution lines developed between hard red winter wheat cultivars Wichita (WI) and Cheyenne, revealed the involvement of chromosome 3A in stomatal resistance and water regulation [90]. Furthermore, recent QTLs mapping and GWAS studies reported that chromosome 3A harbors several QTLs (*QGWt.adh-3A*, *QGWp.adh-3A*,* QGWp.adh-3A*, *Qtgw.cas-3A*, *IWB30485*, *IWA5982*,* QFW_D3A*, etc.) [91–93] and candidate genes (*TaERF-6–3A*) responsible for drought tolerance [20, 92, 94, 95]. Taking together, these findings highlight chromosome 3A as a key genomic region for improving drought tolerance in tetraploid wheat.

### QTL mapping identified a major locus on 4BS from cultivated durum wheat, Altar 84

In this study, we identified a major drought tolerance QTL, *QDt.fcu-4B*, on short arm of chromosome 4B using a RIL population. The short arm of chromosome 4B has previously been reported to harbor QTL associated with drought tolerance in both hexaploid and tetraploid wheat in multiple studies [96–101]. These studies identified multiple QTLs explaining 14.3 to 22% (*qDT4B*,* qDSI.4B.1*, etc.) phenotypic variation for different drought related traits, signifying the importance of this region in genetic control of drought tolerance [65, 102]. Studies also reported multiple strong and stable meta-QTLs under drought stress with large effects on phenotype from this region. For instance, at least five meta-QTLs (*MQTL4B.1* to *MQTL4B.5*) within the 23.6 to 171.6 Mb genomic region were identified on chromosome 4B [103]. Similarly, a major effect meta-QTL, *MQTL1.4*, identified in rice under drought stress was syntenic to this region in wheat [104]. Additionally, proteomic analysis using near-isogenic lines under drought stress identified differentially expressed proteins encoded by genes on the short arm of chromosome 4B, which were mainly involved in reactive oxygen species (ROS) scavenging, oxidative stress response, hydrogen peroxide metabolism, photosynthetic activity, etc [105]. Recently, a group identified a QTL, *qDT4B* (66.8–68.4 Mb region) and cloned a drought tolerance gene, *TaWD40.4B.1*, from this region in hexaploid wheat [65]. This gene encodes a WD40-repeat protein. Compared to tolerant allele, the sensitive allele of this gene had total four SNPs, one in the 3’ UTR (SNP1457) and remaining three in the coding region. Two (SNP465 and SNP999) of the three SNPs in coding region were synonymous and SNP1274 was nonsense and caused the substitution of Trp codon TGG to stop codon TAG. This resulted in a truncated protein in the drought sensitive genotypes. Since, drought tolerance QTLs identified in this study was also located in same genomic region, we speculated that the drought tolerance from Altar 84 might also governed by *TaWD40.4B.1*. Comparing complete gene sequence of the *TaWD40.4B.1* orthologues from Altar 84 and Langdon 16 revealed the presence of non-sense mutation in both genotypes, which strongly suggests that the *TaWD40-4B.1* is non-functional in both lines. Additionally, 100% identity in gene sequence between Altar 84 and Langdon 16 further suggests that drought tolerance in Altar 84 is not controlled by the *TaWD40-4B.1* ortholog. Therefore, the QTL, *QDt.fcu-4B*, identified in this study is a distinct and the drought tolerance in Altar 84 is governed by a different genomic region.

## Conclusion

This study identified two independent drought tolerance loci on chromosome 3A (from PI 481521) and short arm of chromosome 4B (from Altar 84) originated from different donor parents. Currently, we are making biparental mapping populations between LDN-DIC 3A(521) and Langdon 16 to map QTLs on chromosome 3A. Similarly, we are developing a large mapping population by crossing Altar 84 and Langdon 16 with the aim to fine map *QDt.fcu-4B*. Once the QTL region is reduced, opportunity existed for future studies to develop breeder friendly molecular markers for accelerated deployment of these QTLs into breeding programs. Overall, these efforts help us to understand the genetic and molecular mechanisms underlying drought tolerance and will facilitate the development of drought resilient durum wheat cultivars with improved yield under water limited environments.

### Limitations of this study

Despite the successful identification of a major QTL associated with drought tolerance, a few limitations of the study must be acknowledged. For instance, the phenotypic evaluation in this study was performed under controlled greenhouse facilities, which may not fully reflect the environmental variations encountered in the field conditions. Similarly, genotypes were evaluated during seedling stage only, therefore, the stability of QTL must be validated at the later developmental stages including flowering and grain filling stages as well as under diverse environmental conditions. The lack of physiological parameters associated with stress response limits the interpretation of observed phenotypic variation. In addition, low marker density within the QTL region results in large QTL interval with reduced resolution. Finally, breeder friendly diagnostic markers suitable for marker-assisted introgression of identified QTL have not been developed. Future studies must focus on adult plant evaluation at different environments, physiological characterization, fine mapping, and developing diagnostic markers to facilitate marker-assisted selection of drought tolerant genotypes.

## Supplementary Information

Supplementary Material 1: Supplementary Figure 1. Detailed protocol used for evaluating parental lines and their populations under drought stress.

Supplementary Material 2: Supplementary Figure 2. Randomized complete block design (RCBD) used for drought stress screening in the present study. G1-G9 represents the experimental lines and ‘B’ represents the border rows/plants.

Supplementary Material 3: Supplementary Figure 3. Polymerase chain reaction (PCR) amplification of the complete gene sequence of TRITD4Bv1G024340 from Altar 84 and Langdon 16 using primers P1_F (forward primer) and RP6 (reverse primer): (a) gradient PCR with temperatures ranging from 55°C to 64°C to determine optimum annealing temperature to amplify TRITD4Bv1G024340; and (b) amplicons of TRITD4Bv1G024340 with ~2300 bp from Altar 84 and Langdon 16 in three replications.

Supplementary Material 4: Supplementary Figure 4. Quantitative trait loci (QTL) mapping of drought stress response in the recombinant inbred line (RIL) population derived from Altar 84 and Langdon 16. The top graph shows the LOD scores (with a LOD cutoff of 3.0), in the middle graph shows additive effects, and in the bottom graph shows phenotypic variation explained (R2). A dashed line indicates a LOD cutoff of 3.0. Dotted lines along the graphs represent the results for replication 1 (WS_R1; red), replication 2 (WS_R2; green), replication 3 (WS_R3; blue), mean data (WS_mean; pink), BLUP values (WS_BLUP; black). A marker highlighted in blue color represents forward cofactor selection in composite interval mapping (CIM) method.

Supplementary Material 5: Supplementary Figure 5. Sequence alignments showing point mutations and identity between: (a) TRITD4Bv1G024340 from Altar 84 and TaWD40-4B.1; (b) TRITD4Bv1G024340 from Langdon 16 and TaWD40-4B.1; and (c) TRITD4Bv1G024340 from Altar 84 and Langdon 16.

Supplementary Material 6: Supplementary table 1. List of germplasm used in this study. Supplementary Table 2. List of primers used to amplify and sequence the complete TRITD4Bv1G024340 gene. Supplementary Table 3. Raw phenotypic data for wilting score (WS) collected from tetraploid wheat genotypes under drought stress over three replications. Supplementary Table 4. Phenotypic data for wilting score (WS) collected from contrasting genotypes under drought stress. Supplementary Table 5. Raw phenotypic data for wilting score (WS) collected from the PI 478742 chromosome substitution lines in Langdon 16 background under drought stress. Supplementary Table 6. Raw phenotypic data for wilting score (WS) collected from the PI 481521 chromosome substitution lines in Langdon 16 background under drought stress. Supplementary Table 7. Phenotypic data for wilting score (WS) collected from Langdon 16, PI 481521, and a drought tolerant chromosomes substitution line (LDN-DIC 3A(521)) under drought stress over six replications. Supplementary Table 8. Raw phenotypic data for wilting score (WS) collected from recombinant inbred line (RIL) population derived from Altar 84 and Langdon 16 under drought stress.

## Data Availability

The phenotypic datasets generated during the current study are available in the Mendeley Data, [https://data.mendeley.com/datasets/j4tjz4r4wv/1]. The gene sequences generated during the current study are available in the NCBI repository (https://www.ncbi.nlm.nih.gov/bioproject/PRJNA1442025) .
